# Cervical cancer prevention and treatment research in Africa: a systematic review from a public health perspective

**DOI:** 10.1186/s12905-016-0306-6

**Published:** 2016-06-04

**Authors:** Sarah Finocchario-Kessler, Catherine Wexler, May Maloba, Natabhona Mabachi, Florence Ndikum-Moffor, Elizabeth Bukusi

**Affiliations:** 1 Department of Family Medicine, University of Kansas Medical Center, Kansas City, USA; 2 Family AIDS Care and Education Services, Kenya Medical Research Institute, Kisumu, Kenya; 3 Department of Preventive Medicine and Public Health, University of Kansas Medical Center, Kansas City, USA; 4 Center for Microbiology Research, Kenya Medical Research Institute, Nairobi, Kenya; 5 Department of Obstetrics and Gynecology, University of Nairobi, Nairobi, Kenya

**Keywords:** Cervical cancer, Africa, Systematic review, Prevention, Treatment, Quality of life, Feasibility challenges

## Abstract

**Background:**

Women living in Africa experience the highest burden of cervical cancer. Research and investment to improve vaccination, screening, and treatment efforts are critically needed. We systematically reviewed and characterized recent research within a broader public health framework to organize and assess the range of cervical cancer research in Africa.

**Methods:**

We searched online databases and the Internet for published articles and cervical cancer reports in African countries. Inclusion criteria included publication between 2004 and 2014, cervical cancer-related content pertinent to one of the four public health categories (primary, secondary, tertiary prevention or quality of life), and conducted in or specifically relevant to countries or regions within the African continent. The study design, geographic region/country, focus of research, and key findings were documented for each eligible article and summarized to illustrate the weight and research coverage in each area. Publications with more than one focus (e.g. secondary and tertiary prevention) were categorized by the *primary* emphasis of the paper. Research specific to HIV-infected women or focused on feasibility issues was delineated within each of the four public health categories.

**Results:**

A total of 380 research articles/reports were included. The majority (54.6 %) of cervical cancer research in Africa focused on secondary prevention (i.e., screening). The number of publication focusing on primary prevention (23.4 %), particularly HPV vaccination, increased significantly in the past decade. Research regarding the treatment of precancerous lesions and invasive cervical cancer is emerging (17.6 %), but infrastructure and feasibility challenges in many countries have impeded efforts to provide and evaluate treatment. Studies assessing aspects of quality of life among women living with cervical cancer are severely limited (4.1 %). Across all categories, 11.3 % of publications focused on cervical cancer among HIV-infected women, while 17.1 % focused on aspects of feasibility for cervical cancer control efforts.

**Conclusions:**

Cervical cancer research in African countries has increased steadily over the past decade, but more is needed. Tertiary prevention (i.e. treatment of disease with effective medicine) and quality of life of cervical cancer survivors are two severely under-researched areas. Similarly, there are several countries in Africa with little to no research ever conducted on cervical cancer.

## Background

Cervical cancer is the second most common cancer among women worldwide, with an estimated 528,000 new cases and 266,000 deaths among women each year [1]. A disproportionate number of these cases (85 %) and deaths (87 %) occur among women living in low and middle income countries [1]. Women living with HIV are at increased risk of developing cervical cancer [2–4] and experience more rapid progression of the disease [5–7]. Since 1993, cervical cancer was classified as an AIDS-defining illness [8].

The World Health Organization (WHO) advocates a comprehensive approach to cervical cancer prevention and control to identify opportunities to deliver effective interventions [9]. Cervical cancer-related research has increased significantly over the past decade, representing biomedical, behavioral, and policy level findings. Existing review papers synthesize knowledge and advancements for multiple areas of focus within the larger effort of cervical cancer prevention and treatment, e.g., biomarkers for cervical cancer [10, 11], HPV vaccination for young adolescent women [12–15], and feasible approaches to screen and treat adult women in low resource settings [16–20]. The purpose of this systematic review is to assess and characterize recent research within a broader public health framework, utilizing well-known public health terminology to organize and assess the range of efforts to respond to cervical cancer. In this context, these include: Primary Prevention (preventing the initial onset of cervical cancer), Secondary Prevention (early detection by screening and treatment of precancerous cervical lesions), Tertiary Prevention (treatment of cervical cancer to reduce morbidity and mortality), and Quality of Life (post-treatment care or palliative care for those without treatment options) among women in African countries. Literature highlighting feasibility considerations (accessibility, affordability, health care infrastructure, and provider training) and findings specific to HIV-infected women are integrated as appropriate in each public health category.

### Primary prevention

Vaccination is one of the most commonly used public health strategies to reduce the risk of infection and minimize the prevalence of the disease-causing agent (HPV) in the environment. Nearly all cases (99.7 %) of cervical cancer are caused by human papillomavirus (HPV) [21], particularly types 16 and 18 which cause more than two-thirds of all precancerous cervical lesions and cervical cancers [22, 23]. HPV is one of the most common sexually transmitted infections, with up to 75 % of sexually active people estimated to be infected at some point during their lives [24]. Fortunately, two vaccines are approved for use. The bivalent vaccine protects against HPV types 16 and 18. The quadrivalent vaccine protects against HPV types 16 and 18 and also types 6 and 11, which cause 90 % of genital warts [25]. Since HPV infection often occurs shortly after the onset of sexual activity (over 35 % of women are infected within 2 years of initiating sexual activity) [26–28], vaccination campaigns should target 9–13 year old youth, prior to sexual debut. The vaccines are over 95 % effective at preventing HPV infection caused by vaccine-type HPV when the full three course dose is given over six months [29, 30]. Since 2014, the WHO recommends a two-dose regimen for girls and boys aged 9–13 (quadrivalent vaccine) or aged 9–14 (bivalent vaccine) [31], which is not yet licensed in all countries, but reduces the follow-up burden while maintaining strong protective coverage [32–35].

The WHO recommends the inclusion of HPV vaccination in national immunization programs provided HPV represents a public health priority and vaccine delivery is feasible and cost-effective [31]. Unfortunately, HPV vaccination is not yet available in many African countries. By August 2014, only 58 countries had introduced HPV vaccination for girls into their national immunization program [31]. While the majority of these are high-resource countries, a few low to middle income countries in Africa including Rwanda, South Africa, Lesotho, and Uganda [36] have also introduced national HPV vaccines. In 2013, the Global Alliance for Vaccines and Immunizations (GAVI) began providing support for HPV vaccinations to eligible countries and will support demonstration projects in 23 countries, of which ten have been launched, primarily in sub-Saharan Africa [37, 38]. However, barriers to vaccination (i.e. concerns about the safety of the vaccine, provider reservations about recommending vaccination for younger girls, limited awareness of the relationship between HPV and cervical cancer, and varied parental acceptance of the HPV vaccine [39–42]) result in inconsistent vaccine uptake, globally [13, 43]. Recent efforts to vaccinate young boys have received less focus, but may help indirectly protect girls by reducing the risk of re-infection with HPV and will help prevent other HPV-related morbidities for men including penile cancer, anal cancer, oropharyngeal cancer, and genital warts [44].

Other primary prevention strategies to reduce HPV infection and cervical cancer include delaying sexual debut, reducing the number of lifetime sexual partners, and increasing condom use [45]. In addition to reducing HIV acquisition and transmission, medical male circumcision is also protective for HPV in males [46–48], which reduces the risk of initial or re-infection of HPV among women.

### Secondary prevention

Screening for early detection and treatment is a cornerstone of secondary prevention. Early diagnosis and treatment of cervical pre-cancerous lesions prevents up to 80 % of cervical cancers in high resource countries where cervical cancer screening is routine [49]. In higher income countries, cervical cytology (Pap smear) in which cervical cells are examined in order to detect cervical intraepithelial neoplasia (CIN) became part of routine care in the 1940’s [50]. For women who screen positive for premalignant cervical lesions (i.e., CIN), a confirmatory colposcopy is required [51]. Cervical cytology, however, is not a feasible method of screening in many African countries given the required level of medical and laboratory infrastructure and trained personnel, multiple return visits with poor patient tracking strategies, and availability of such services often limited to capital cities. The proportion of women in sub Saharan Africa reporting a pelvic exam and pap test in the previous three years is very low (1.0 % in Ethiopia to 23.2 % in South Africa), with 40 % of women in Tunisia to 94 % of women in Malawi having never received a pelvic exam [52, 53].

The more feasible, and WHO-approved, strategy for cervical cancer screening in low resource settings is visual inspection with acetic acid (VIA) or visual inspection with Lugol’s iodine (VILI). After applying acetic acid or Lugol’s iodine directly on the cervix, pre-cancerous and cancerous lesions turn white, making them visible to the naked eye [54]. This method has high sensitivity among HIV-infected and uninfected women [55, 56]. Results are immediate, thus women who screen positive for precancerous lesions can theoretically be offered cryotherapy treatment during the same visit, or a “screen and treat” approach, if the health facility has the capacity. This strategy has been shown cost-effective, affordable, and an ideal first-line treatment for CIN of any grade when the cervical lesion size and location allows the cryoprobe tip to make adequate contact [57–60]. This ‘screen and treat’ strategy can avoid the burden of costly follow up visits, significant delays in treatment, and loss to follow up [61, 62].

Cryotherapy can be performed at the primary care level by mid-level providers, such as nurses or midwives, who can be trained to perform cryotherapy with a minimum of supplies and equipment [58, 63]. In developed countries, cryotherapy is approximately 90 % effective for all grades of CIN after 1 year [64]. The most commonly employed treatment options for pre-cancerous lesions are removal of diseased tissue using loop electrosurgical excision procedure (LEEP) which requires local anesthetic [65, 66] or by freezing the affected tissue with cryotherapy [57]. These treatments are typically performed by trained providers in outpatient clinics at provincial or referral level hospitals. Cold-knife conization [65] can be used to remove lesions that cannot be effectively treated with LEEP or cryotherapy.

The optimal frequency of screening is every 3–5 years depending on screening method [67], or within 3 years for women living with HIV [68]. If a woman can be screened only once in her lifetime, the most strategic age is between 30 and 39 years [69]. Recent estimates on national rates of cervical cancer screening are not available for many African countries, but a number of studies report self-reported screening rates to be low (8.3–64 %) [70–74], but slightly higher among women accessing HIV-care (9.4–80 %) [72, 75, 76]. A 2008 population-based survey in 57 countries estimated 19 % of women in developing countries were screened for cervical cancer in the preceding three years [77]. In addition to infrastructure and resource-related barriers, awareness of cervical cancer among reproductive-aged women remains low [70, 78–81] and an inadequate proportion of health care providers has been trained to provide high quality screening [82, 83]. Efforts have been made in many countries to integrate cervical cancer screening in HIV care, but routine provision is still limited [77, 84]. HPV DNA testing represents an emerging strategy for early detection of cervical cancer, particularly if technology innovations can permit point-of-care testing which will eliminate requirements for laboratory infrastructure and technical support [68, 85]. HPV DNA testing must be made affordable for widespread use in African countries.

### Tertiary prevention

Women with abnormal cervical tissue are diagnosed with either precancerous lesions or invasive cervical cancer, both of which require treatment. Severe cervical dysplasia that remains undiagnosed or untreated can develop into invasive cancer [86]. Unfortunately, a significant proportion of women (56–80.6 %) [(Kenya) [87]; (Tanzania) [88]; (Nigeria) [89]) are identified once their cervical cancer is at an advanced stage [90].

Staging the severity of invasive cervical cancer requires assessment of the vagina, parametrium, urinary bladder and rectum by a combination of clinical and endoscopic procedures to determine the stage of progression (I – IVB) [91]. Inadequate laboratory facilities and personnel shortages may result in treatment decisions being made without proper diagnoses or adequate information. Treatments for invasive cervical cancer can include a range and combination of strategies including hysterectomy (requires surgical facilities), radiotherapy (external and intracavitary radiotherapy infrastructure), and chemotherapy [68, 92, 93]. The availability of these options are typically limited to capital cities in several African countries or, in some cases, not available at all [94]. Consequently, palliative care with symptom control and support may be the most likely option for severely late stage cervical cancer or for women with less advanced disease, but who cannot afford or access treatment. Studies indicate that only between 24–67 % of those diagnosed with cervical cancer in Tanzania, Zimbabwe, Uganda or Nigeria received some form of treatment (either radiotherapy or hysterectomy) [88, 89, 95, 96], with women in advanced stages (III and IV) of disease progression [96] and women co-infected with HIV [88] less likely to be treated.

Cervical cancer mortality rates in low resource countries are nearly three times as high as rates experienced in high resource settings [1, 97, 98]. Survival data for cervical cancer in African countries are limited. Estimated 5-year survival for women diagnosed with cervical cancer in 7 African countries between 2005 – 2009 was 56.3 % (range 19.5–96 %) [99]. Among women receiving treatment (radiotherapy and/or surgery), survival probabilities at one year post diagnosis ranged from 73.9–90.4 % and decreased progressively to 32.5 % by four years [87, 95, 96, 100]. Without treatment, observed survival is 58.6 at 1 year and decreases to 31.1 % by 4 years [95, 96]. Studies from Uganda and Zimbabwe suggest that although treatment with radiotherapy improves patient survival two to three years after diagnosis, this advantage disappears in later years [95, 96].

### Quality of life

Cervical cancer is associated with psychological and physical morbidities that negatively impact quality of life [101, 102]. Age-adjusted, daily-adjusted life years (DALY) lost from cancer in African countries is consistently higher than those of high resource countries [103]. The estimated DALY lost from cervical cancer in sub-Saharan Africa is 641 years per 100,000 women [103]. Quality of life is most compromised among patients with inoperable cervical cancer treated by radiotherapy, with a majority reporting deterioration in physical, emotional, social, and economic support [104], and the highest risk for long-term dysfunction of bladder, bowels and psychosocial consequences [105]. Other treatment related side-effects such as extended vaginal bleeding and chronic radiation enteritis can affect physical and social aspects of their quality of life [106, 107].

Significant changes in the sexual domain resulting in marital discordance [104] and waning partner support over the course of treatment and survival [108] have been reported in Kenya and South Africa. While such data are limited for low-resource settings, literature reviews from high resource countries document post-treatment changes in body image, vaginal function, sexual satisfaction, and sexual relationship with partner; indicating a clear need for better integration of sexuality rehabilitation into routine clinical care [109–111]. Furthermore, there are few treatment options available that preserve fertility [92, 112–114], which can have significant implications for young women given the personal and cultural importance of childbearing [115]. Open communication about fertility and sexuality-related issues with cervical cancer patients of reproductive-age should occur prior to treatments to help shape expectations and quality of life during recovery [113].

In most African countries, there is a long historical precedent of providing palliative care at home by family or community members [116]. Although strengthened by the AIDS response, palliative care efforts still fail to provide effective pain relief [91], with the availability and accessibility of opioids for pain relief severely limited in African countries [117]. In a study among Nigerian cancer patients (including cervical cancer patients), the presence of pain was significantly associated with depressive and anxiety symptoms, suicidal ideation, poor sleep, impaired concentration, lack of opportunity for leisure, dissatisfaction with health, poor overall quality of life, poor ability to get around and the need for excessive medical treatment to function in daily life [118]. In 2011, only four African countries had integrated palliative care into their cancer strategic plans and two others had stand-alone national palliative care policies [119]. Pain management for cancer patients should not be neglected as countries develop and adapt their response to cervical and other cancers.

### Feasibility considerations

Governments grapple with challenges posed by limited funds and competing healthcare priorities including a heavy burden from both infectious and chronic diseases. While the African Cancer Registry Network (AFCRN), launched in 2012, supports 25 cancer registries in 19 member countries in sub Saharan Africa [120, 121], many still lack established cancer prevention and control health policies [94, 122]. The geographic distribution of cancer treatment centers with cytology laboratories, radiotherapy and chemotherapy infrastructure severely limits accessibility for residents in more rural areas [49, 54, 85]. A 2009 situational analysis of east, central, and southern African countries estimated that only 4 % of institutions had equipment to perform outpatient treatment modalities such as cryosurgery [123]. This demand for scarce services typically results in long waiting periods (median 3.8 months in Ethiopia [100]) for treatment. The costs for return hospital visits, pathology reports, and subsequent treatment are beyond the resources of the majority of women in these settings [124]. According to a recent study, infrastructure investment of approximately $59 million would be required to equip every cervical cancer screening facility with cryotherapy equipment in 23 high-incidence sub-Saharan African countries [125] to be able to employ the recommended screen and treat approach [51]. Estimating the ability to screen nearly 20 million women over a 10 year period in these targeted countries, the costs for screening (VIA) would be less than $10 USD per woman, and the costs for treatment by cryotherapy or LEEP would range between $38 to $71 USD per woman [125]. Systems to track and refer women who test positive and support treatment retention are also lacking; diminishing the return on investment of current screening efforts.

## Methods

We searched several online databases including PubMed/MEDLINE (NCBI), Embase (Elsevier), African Index Medicus (AIM), and Google Scholar for published studies. Our search also included highly relevant global and government reports not published in peer-reviewed journals. Inclusion criteria included publication between 2004 and 2014, cervical cancer-related content pertinent to one of the four public health categories (primary, secondary, tertiary prevention or quality of life), and conducted in or specifically relevant to countries or regions within the African continent. Reference sections of articles were reviewed to identify additional eligible articles. Searches for each of the four focus areas were conducted separately, including combinations of the following search words: 1) primary prevention [*HPV vaccination, Africa, cervical cancer prevention, HPV prevention, cost effectiveness, medical male circumcision, coverage*], 2) secondary prevention [*cervical cancer screening, Africa, cervical cancer secondary prevention, HPV screening, screen and treat, male HPV screening*], 3) tertiary prevention [*cervical cancer treatment, Africa, SSA, pre-cancerous lesions, invasive cervical cancer, management of invasive cervical cancer in Africa, cervical cancer survival, HIV, access to treatment, challenges,*], and 4) quality of life [*cervical cancer, quality of life, Africa, sexual function, palliative care, relationship challenges, partner support, treatment recovery*]. A separate search was conducted to identify research focused on issues of feasibility and infrastructure [*health care infrastructure for cervical cancer, Africa, accessibility, affordability, training health care providers, provider knowledge, feasibility, HPV vaccine, screening, screen and treat, treatment*]. Rather than creating a section dedicated to feasibility issues, these were integrated into the preceding categories, depending on the focus of the article.

The study design, geographic region/country, focus of research, and key findings were documented for each eligible article. Articles and reports were maintained in four separate spreadsheets (reflecting the four priority categories) and later combined to review and eliminate any duplication and to determine the best grouping for articles that may have covered more than one primary category. The articles and reports under each primary category were then analyzed and refined to highlight sub-themes, type of literature (original research, review article, lessons learned or policy paper) and the country or geographic region of research, Tables 1, 2, 3 and 4.

**Fig. 1:**
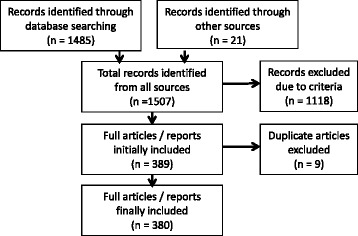
PRISMA flow diagram of the number of searches yielded, excluded, and reviewed

One thousand five hundred six records were reviewed through database and Internet searches (Fig. 1). Among those 389 met the criteria for inclusion. After eliminating nine duplicates among the categories, a total of 380 records were included in this review. Publications have been organized according to four targeted categories of public health interest to illustrate the weight and coverage of research in each area. Publications that span more than one area of focus (e.g. secondary and tertiary prevention) were categorized by the *primary* emphasis of the paper.

This systematic review did not require approval from an ethical review board or informed consent with participants as research efforts were limited to the review and analysis of previously published or presented cervical cancer research in African countries.

## Results

### Primary prevention

Table 1 reports the *predominate* focus of each article describing primary prevention. Of all 89 publications identified, 76 (85 %) focused on the HPV vaccination for the prevention of HPV/cervical cancer. Only 13 (15 %) focused on other prevention methods (mainly male circumcision but also condom use and microbicides), of which eight were performed in Uganda by the same study group. We could not find any study that examined the prevention of HPV and/or cervical cancer by delayed sexual debut, abstinence, and/or limiting the number of sexual partners. Considering the multiple foci of each paper, rather than just the primary focus, 17 talked about the cost-effectiveness or estimated impact of HPV vaccinations; 40 had a focus on acceptability, knowledge, and/or attitudes [12, 39–41, 126–152]; ten had a focus on uptake and/or retention [43, 152–160]; five had a focus on populations affected by HIV [47, 161–163]; four had a focus on males [132, 138, 150, 158]; four had a focus on safety and immunogenicity [161, 162, 164, 165]; one on adverse events [166]; and three on policy [167–169]; ten on male circumcision [46–48, 170–176]; two on lubricants, gels, and/or microbicides [177, 178], one on diaphragms [177]; and one on condoms [179].

**Table 1:** Primary Prevention Research, *n* = 89

Focus			Literature type	Countries/Geographic Regions Included
Primary Prevention	HPV Vaccination	Knowledge/Acceptability/Attitudes (*N* = 29)	25 Original research [39–41, 126, 127, 130–132, 134–137, 139, 144–151, 158, 180, 256, 257] 2 Review Articles [12, 15] 0 Lessons Learned 2 Policy Papers [167, 258]	Tanzania [126, 180], Uganda [40, 127], Nigeria [131, 137, 256], South Africa [130, 139, 148, 150, 151, 158], Malawi [41], Mali [132, 147], Cameroon [134, 144], Kenya [39, 145, 257], Botswana [135], Ghana [136], Zambia [146], Morocco [149]
		Uptake/Retention (*N* = 10)	6 Original research [43, 153, 155, 157, 159, 160] 1 Review Articles [259] 3 Lessons Learned [154, 156, 260] 0 Policy	Uganda [153, 259, 261], Lesotho [43, 155], Cameroon [43, 155, 156], Tanzania [155, 160], Uganda [155, 157], Kenya [155], South Africa [159], Rwanda [154, 260]
		Feasibility (*N* = 23) Cost/cost effectiveness [14, 170, 181, 214, 261–269] Estimated impact [270–272] Provider knowledge/training [128, 129, 133, 140–143]	21 Original research [128, 129, 133, 140–143, 170, 181, 214, 261, 263–272] 2 Review Articles [14, 262] 0 Lessons Learned 0 Policy	Tanzania [14, 181, 267, 269], Uganda [14, 267, 271], Sub-Saharan Africa [262, 264], Mali [263, 270], Nigeria [128, 140, 141, 143, 265], South Africa [129, 142, 214], GAVI-eligible countries [266, 268, 272], Kenya [267, 271], Mozambique [271], Zimbabwe [271], Guinea [170], Cameroon [133]
		HIV (*N* = 2)	2 Original research [161, 162] 0 Review Articles 0 Lessons Learned 0 Policy	South Africa [161, 162]
		Males (N = 1)	1 Original research [138] 0 Review Articles 0 Lessons Learned 0 Policy	Uganda [138]
		Other (*N* = 11) Safety/Immunogenicity (*n* = 2) [164, 165] Adverse Events (n = 1) [166], Barriers (*n* = 1) [42], Policy (*n* = 6) [168, 169, 273–276], Conference Summary (*n* = 1) [152]	3 Original research [164–166] 2 Review Articles [42, 152] 0 Lessons Learned 6 Policy [168, 169, 273–276]	Uganda [166, 168, 274], Senegal [164], Tanzania [164, 165], Low-Middle Income Countries [42, 273, 276], Middle East/North Africa [275], Sub-Saharan Africa [152, 169]
		Primary Prevention (HPV Vaccine) Total = 76	59 Original research 6 Review Articles 3 Lessons Learned 8 Policy	South Africa 12, Uganda 12, Tanzania 10, Nigeria 8, Cameroon 6, Kenya 6, Mali 4, Low-Middle Income Countries 3, GAVI Eligible Countries 3, Lesotho 2, Rwanda 2, sub-Saharan Africa 4, Malawi 1, Botswana 1, Ghana 1, Zambia 1, Morocco 1, Ivory Coast 1, Mozambique 1, Zimbabwe 1, Middle East/North Africa 1, Guinea 1
	Non-Vaccine Prevention	Male Circumcision (*N* = 10)	10 Original research [46–48, 171–176, 277] 0 Review Articles 0 Lessons Learned 0 Policy	Uganda [47, 48, 171–174, 176, 277], South Africa [46, 175]
		Condoms (*N* = 1)	1 Original research [179] 0 Review Articles 0 Lessons Learned 0 Policy	South Africa [179]
		Lubricant Gel, Microbicides and/or Diaphragm (*N* = 2)	2 Original research [177, 178] 0 Review Articles 0 Lessons Learned 0 Policy	South Africa [178], Zimbabwe [177]
		Primary Prevention (Non-Vaccine) Total = 13	13 Original research	Uganda (8), South Africa (4), Zimbabwe (1)

Of the 76 publications about HPV vaccination, the researchers and/or study received some support from the pharmaceutical companies that manufacture HPV vaccinations in 16 (21 %) of the studies [43, 126, 142, 153, 155–157, 160–162, 164–166, 170, 180, 181]. All studies that looked at safety/immunogenicity of the vaccines in HIV-infected [161, 162] and HIV-uninfected [164, 165] women were funded by pharmaceutical companies.

Knowledge/awareness and acceptability of the HPV vaccine was often reported together and assessed in many different countries in SSA and among many different populations. While acceptability of HPV vaccination tends to be high, knowledge and awareness of the vaccination is low, even amongst health care workers. There is a glaring lack of literature regarding HPV vaccination among HIV-positive populations and among males. Despite some data indicating HPV’s role in other cancers (i.e. penile, anal, throat and mouth), all vaccination programs have focused solely on cervical cancer prevention. The few studies that focused on males emphasized their ability to influence HPV vaccine decision making in females or HPV vaccination in males to help protect females from cervical cancer. Direct benefits for men’s health (i.e. reductions in genital warts, and penile, anal, throat/mouth cancers) were not highlighted.

Geographically, South Africa and Uganda contributed the most research, representing 18 and 23 % of all primary prevention articles, respectively, and were represented in both vaccination and non-vaccination articles.

### Secondary prevention

We identified a significant number of publications (*n* = 208) focused on various aspects of secondary prevention of cervical cancer. Table 2 reports the *predominant* focus of each article. The primary focus of the 208 published articles include: 46 (22 %) on screening test performance; 16 (8 %) on screen-and-treat program implementation and/or outcomes; 28 (13 %) on screening feasibility including infrastructure, provider knowledge and attitudes, and costs; 59 (29 %) on women’s knowledge about, attitudes towards, and uptake of cervical cancer; 18 (8 %) on cervical cancer screening specifically among HIV+ women; 3 (1.5 %) on aspects of HPV/cervical cancer screening relevant to males; 23 on program (excluding screen-and-treat programs) or intervention implementation and/or outcomes; and 15 focused on other categories. A total of 65 (32 %) reported on observed or self-reported uptake (ranging between 0–100 %; mean = 25 %; median = 15 %) among different populations

**Table 2:** Secondary prevention research, *n* **=** 208

	Primary focus	Literature type	Countries/Geographic Regions Included
Secondary Prevention	Screening Test Performance (*N* = 46) 11 VIA/VILI [194, 196, 197, 232, 278–284] 15 HPV Testing [163, 182–192, 285–287] 3 Cytology [288–290] 1 Colposcopy [291] 16 Comparison of 2+ tests [16, 193, 195, 198, 292–303]	41 Original Research [182, 184–186, 188–198, 232, 278–293, 295, 296, 298–303] 4 Review Article [16, 187, 294, 297] Lessons Learned 1 Policy Papers [163]	Africa (2) [285, 297], Developing Countries (4) [16, 284, 294, 302], Angola (1) [197], Botswana (1) [232], Burkina Faso (5) [194, 280, 282, 291, 298], Cameroon (4) [182, 193, 286, 300], Democratic Republic of Congo (3) [195, 198, 296], Republic of the Congo (6) [191, 194, 280, 282, 291, 298], Egypt (2) [186, 278], Gambia (1) [192], Ghana (1) [288], Guinea (5) [194, 280, 282, 291, 298], Ivory Coast (1) [190], Kenya (2) [184, 301], Mali (5) [194, 280, 282, 291, 298], Niger (5) [194, 280, 282, 291, 298], Nigeria (4) [279, 287, 289, 293], Rwanda (1) [294], South Africa (7) [163, 183, 187, 188, 290, 292, 299], Tanzania (2) [196, 295]
	Screen-and-Treat (SNT) (*N* = 16) 8 VIA then Cryotherapy [63, 199–201, 304–307] 5 Other SNT methods [61, 308–311] 3 Guidelines/Overview [51, 202, 203]	10 Original Research [61, 63, 200, 201, 305, 307–311] 2 Review [203, 304] 2 Lessons Learned [199, 202] 2 Policy [51,283 1,217]	Global (1) [51], Africa (1) [203], Botswana (1) [307], Ghana (1) [63], Kenya (1) [61], Madagascar (1) [306], Malawi (1) [306], Nigeria (2) [306, 311],South Africa (2) [308, 310], Tanzania (1) [306], Uganda (2) [200, 306], Zambia (7) [199, 201, 202, 304–306, 309]
	Feasibility (*N* = 28) 2 Infrastructure [215, 220] 18 Provider Knowledge/Training/Attitudes [60, 82, 83, 208–211, 312–322] 8 Cost/Cost-effectiveness [59, 125, 212–214, 323–325]	26 Original Research [59, 83, 125, 208–215, 220, 312–325] 2 Review [60, 82] 0 Lessons Learned 0 Policy	Africa (3) [82, 125, 212], Cameroon (1) [313], Ghana (1) [323], Ivory Coast (1) [208], Kenya (2) [59, 325], Nigeria (11) [83, 211, 220, 312, 315–317, 319–321], South Africa (6) [59, 213, 214, 318, 324, 325], Tanzania (1) [209], LRC [60]
	Awareness and Utilization (*N* = 59) 44 Knowledge, Attitudes, Uptake [53, 70, 72, 74, 78–80, 204–207, 225, 326–357] 15 Determinants of Uptake [62, 73, 358–370]	59 Original Research [53, 62, 70, 72–74, 78–80, 204–207, 225, 326–370] 0 Review 0 Lessons Learned 0 Policy	Global (1) [368], Developing Countries (1) [328], Botswana (3) [72, 205, 349], Cameroon (1) [74], Ghana (2) [70, 326], Kenya (5) [204, 335, 340, 355, 358], Malawi (1) [53], Mozambique (1) [207], Morocco (1) [365], Nigeria (18) [78, 79, 225, 332–334, 337, 338, 342, 344–346, 348, 351, 352, 363, 366, 367], South Africa (12) [80, 327, 329, 330, 341, 347, 353, 356, 360, 361, 369], Sudan (1) [343], Tanzania (5) [62, 336, 362, 364, 370], Tunisia (1) [331]
	HIV-Positive Women (*N* = 18) 5 Service Integration [77, 84, 229–231] 9 Test Performance [371–379] 3 Knowledge, Attitudes, Uptake [75, 227, 228] 1 Screen-and-Treat [226]	17 Original Research [75, 77, 84, 226–230, 371–379] 1 Review [231] 0 Lessons Learned 0 Policy	Global (1) [231], Burkina Faso (1) [373], Cameroon (1) [230], Kenya (4) [229, 374, 376, 378], Nigeria (3) [75, 84, 227], South Africa (6) [226, 228, 371, 373, 375, 377], Uganda (1) [379], Tanzania (1) [77]
	Males and HPV Screening (*N* = 3) 1 Knowledge and Beliefs [221], 1 Male Partner Support [222], 1 Penile HPV Detection [223]	3 Original Research [221–223] 0 Review 0 Lessons Learned 0 Policy	Ghana (1) [221], Kenya (1) [223], Uganda (1) [222]
	Other (*N* = 38) 23 Program/Policy Implementation [54, 71, 216–219, 224, 380–395] 3 Screening Follow-Up [396, 397] and Side-effects [398] 5 Overview of Current Situation [123, 399–402] 7 Other Ethical Issues,[403] message development, [404] overviews,[69, 85, 405, 406] publication output [407]	17 Original Research [71, 216, 217, 219, 224, 380, 381, 383–385, 388, 395–398, 403, 404] 14 Review [54, 69, 85, 123, 382, 391, 392, 394, 399, 400, 402, 405–407] 4 Lessons Learned [218, 386, 387, 393] 3 Policy [389, 390, 401]	Africa (3) [391, 400, 405], Developing Countries (7) [54, 69, 85, 123, 382, 392, 406], Middle East/North Africa (1) [399], Ghana (2) [217, 401], Ivory Coast (1) [224], Kenya (2) [384, 403], Mali (1) [383], Mozambique (1) [216], Nigeria (6) [386–388, 393, 396, 398], South Africa (11) [71, 218, 219, 381, 385, 390, 394, 395, 402, 404, 407], Sudan (1) [389]
	Total *N* = 208	173 Original Research 23 Review Article 6 Lessons Learned 6 Policy Papers	Global (3), Africa (9), Developing Countries (11), Middle East/North Africa (1), Angola (1), Botswana (5), Burkina Faso (6), Cameroon (7), Congo (9), Egypt (2), Gambia (1), Ghana (8), Guinea (5), Ivory Coast (3), Kenya (17), Madagascar (1), Malawi (2), Mali (6), Morocco (1), Mozambique (2), Niger (5), Nigeria (43), Rwanda (1), South Africa (45), Sudan (2),Tanzania (9), Tunisia (1), Uganda (4), Zambia (7),

.

VIA/VILI and HPV testing were the most commonly evaluated screening tests, both individually and when compared with other tests. Of the 15 articles examining the performance of HPV testing, 12 compared different sample collection methods (i.e. self vs. physician collected samples [182–189]; dried cervical spots [190, 191]; tampons [188, 191, 192]). Sensitivity and specificity of each test ranged from 25–91.7 % [193, 194] and 64.6–98.2 % [195, 196] for VIA, 44–97.7 % [194, 195] and 68.9–97.3 % [196, 197] for VILI, 31–90.4 % [193, 195] and 84.5–96.9 % [193, 198] for cytology, and 63.9–100 % [192, 198] and 73–96.6 % [184, 198] for HPV DNA testing, respectively. The sensitivity and specificity of self-collected and physician collected HPV samples were similar, making it useful for women who may be reluctant to undergo a pelvic exam.

Most (53 %) of the articles focusing on a screen-and-treat approach assessed VIA/VILI and then treatment with cryotherapy. While many reported on barriers, challenges, and lessons learned from this approach [199–202], most indicated that screen-and-treat methods were safe, acceptable, and feasible in African settings and reduced loss-to-follow up after a positive screening test. One commentary [203] was critical of screen-and-treat approaches, stating a lack of evidence of safety which can compromise acceptability and potential effectiveness of all screening programs. Articles focusing on awareness and utilization were mostly cross-sectional studies and reported low levels of knowledge and awareness of cervical cancer screening, but generally positive attitudes [204, 205]. This was supported by higher uptake (59.6–100 %) of screening among women who were offered the test as part of an intervention [71, 75, 84, 206, 207].

The majority of articles in the feasibility section focused on provider knowledge, awareness, and acceptability. Although knowledge of cervical cancer and screening methods was higher amongst health care workers compared to the general population [208–211], utilization of screening among female health care workers remained low (4–41 %). Cervical cancer screening programs were extremely cost-effective, either implemented by themselves [59, 212, 213] or in combination with other cervical cancer prevention programs [214]. However, infrastructure challenges reported in both feasibility and program/policy implementation articles (long travel distances to screening and care centers, lack of gynecologists and laboratory pathologists and other manpower shortages, equipment problems, poor record keeping, inadequate patient follow-up, and delayed testing results [215–220]) make wide scale implementation and utilization challenging in many African settings.

Only three articles were identified that focused primarily on males and cervical cancer screening in Africa [221–223]. Similarly, while several articles included HIV-positive women in their analyses [72, 224, 225], only 16 focused exclusively on cervical cancer screening among HIV-positive women. Despite a higher positivity rate among HIV-infected women [224, 226], knowledge of cervical cancer screening [75, 225, 227, 228] and uptake [72, 75, 227, 228] is still low. Integrating HIV care and cervical cancer screening [229–231] and utilizing telemedicine [232] may provide viable methods for providing cervical cancer screening to HIV-positive women.

Geographically, the countries with the most research on secondary prevention were South Africa (*n* = 45), Nigeria (*n* = 43), and Kenya (*n* = 17). Countries were not evenly represented by category of secondary prevention. In several cases, a country was well-represented within one category but not in the others. For example, well represented in the screening test performance category were Republic of the Congo (*n* = 6) Guinea (*n* = 5), and Niger (*n* = 5), yet these countries were not represented within any of the other categories.

### Tertiary prevention

We identified *n* = 67 articles highlighting aspects of tertiary prevention of cervical cancer in African countries, reported in Table 3. The primary focus of published studies and reports included: 7 (10 %) on treatment of precancerous lesions, 8 (12 %) on diagnosis and staging, 6 (9 %) on treatment for invasive cervical cancer, 8 (12 %) on survival outcomes, 23 (34 %) on HIV and cervical cancer, and 14 (21 %) regarding the feasibility of providing cervical cancer treatment. Treatment programs for cervical cancer are limited in most countries in Africa, as is research contributing outcome data, lessons learned, and implementation recommendations. Late diagnosis was routinely documented in several African countries among both HIV+ and HIV-uninfected women, with the proportion diagnosed with stage III or higher ranging from 56 to 90 % [87, 88, 233, 234]. Suboptimal management of symptomatic patients (in frequent gynecologic exams and appropriate referrals) further exacerbates the challenge of late diagnosis [234].

**Table 3:** Tertiary prevention research, *n* = 67

Focus		Literature type	Countries/Geographic Regions Included
Tertiary Prevention	Treatment of Pre-Cancerous lesions 3 LLETZ [408–410] 2 Cryotherapy [57, 411] 2 Comparison LEEP/Cryotherapy [311, 412] *N* = 7	5 Original research [311, 408–411] 2 Review Articles [57, 412] 0 Lessons Learned 0 Policy	Kenya (1) [411], South Africa (3) [408–410], SSA (2) [57, 412], Nigeria (1) [311]
	Late Diagnosis/Staging *N* = 8	8 Original research [88, 233, 234, 365, 413–416] 0 Review Articles 0 Lessons Learned 0 Policy	Nigeria (3) [413–415], Morocco (1) [365], Tunisia (1) [416], Zimbabwe (1) [233], Tanzania (1) [88], South Africa (1) [234]
	Treatment of Invasive Cervical Cancer 2 Surgery [417, 418] 3 Radiotherapy [419–421] 2 Combination therapy [422, 423] *N* = 7	5 Original research [417, 418, 420, 421, 423] 1 Review Articles [422] 1 Lessons Learned [419] 0 Policy	SSA (1) [422], South Africa (3) [418, 420, 423], Uganda (1) [421], Senegal (1) [419], Nigeria (1) [417]
	Survival *N* = 8	6 Original research [87, 95, 100, 424–426] 2 Review Articles [427, 428] 0 Lessons Learned 0 Policy	Malawi (1) [424], Uganda (2) [425, 428], Gambia (1) [428], Ethiopia (1) [100], South Africa (1) [426], Zimbabwe (1) [95], Kenya (1) [87], Egypt (1) [427]
	HIV and treatment 8 HIV and CIN [235, 236, 240–242, 244, 429, 430] 8 HIV and ICC [238, 239, 243, 246, 431–434] 7 HIV/Treatment of CIN and ICC [239, 245, 247, 248, 435–437] *N* = 23	22 Original research [235, 236, 238–248, 429–433, 435–437] 1 Review Articles [434] 0 Lessons Learned 0 Policy	South Africa (11) [240, 242, 243, 245, 247, 248, 429, 432, 433, 436, 437], Kenya (5) [235, 236, 239, 244, 430], Uganda (1) [238], Global/SSA (3) [241, 246, 434], Cote ‘Ivoire (1) [431], Senegal (1) [435]
	Feasibility 1 Access/Awareness/Health Policy [255] 6 Training needs [92, 438–442] 5 Infrastructure needs [443–447] 2 Costs [448, 449] *N* = 14	10 Original research 1 Review Articles [445] 0 Lessons Learned 0 Policy Papers 2 Meeting report [92, 442, 447] 1 Book Chapter [255]	Africa (4) [441–443, 447], Kenya (1) [438], Botswana (1) [444], Nigeria (1) [439], SSA (4) [92, 255, 445, 446], Morocco (1) [448], Tunisia (1) [449], South Africa (1) [440]
	*N* = 67	57 Original research 7 Review Articles 1 Lessons Learned 0 Policy Papers 2 Meeting report 1 Book Chapter	Kenya (8), South Africa (20), SSA (10), Nigeria (6), Morocco (2), Tunisia (2), Zimbabwe (2), Tanzania (1), Uganda (4), Senegal (2), Malawi (1), Ethiopia (1), Gambia (1), Egypt (1), Cote d’Ivoire (1), Botswana (1)

The treatment sections for precancerous lesions and invasive cervical cancer are delineated by treatment strategy to indicate areas receiving more or less research focus. Studies describing outcomes or evaluating treatment strategies for precancerous lesions included loop electrical excision procedure (LEEP) vs cryotherapy (*n* = 2), large loop excision of the transformation zone (LEETZ) (*n* = 3), and cryotherapy (*n* = 2). These treatment strategies were found to be well accepted [235, 236]. Studies regarding treatment for invasive cervical cancer included surgery (*n* = 2), radiotherapy (*n* = 3) and combined radiochemotherapy (*n* = 2). This dearth of research reflects the limited capacity for cervical cancer treatment in African countries. According to the African Organization for Research and Training in Cancer (AORTIC) published in 2013, 22 % of the 54 countries in Africa have no access to any form of anti-cancer therapies, which include surgical oncology, chemotherapy and radiation [237]. Among women who do begin treatment, loss to follow up continues to be a problem [61, 238, 239].

The largest proportion of articles was specific to conditions and outcomes of HIV+ women with CIN or cervical cancer, followed by an emphasis on feasibility considerations for providing treatment. HIV+ women were more likely to present with CIN or cervical cancer at an earlier age [240], have higher grade CIN [241, 242], faster progression [243], more likely to experience CIN reoccurrence after treatment [236], and have poorer treatment and survival outcomes [238, 243, 244]. Antiretroviral therapy was shown to improve treatment outcomes for CIN and cervical cancer [245–248]. While feasibility articles were more comprehensive in nature, we categorized them according to the primary feasibility issue discussed: training needs of health care personnel on cervical cancer management (*n* = 6), the lack of medical infrastructure and scarcity of equipment to provide treatment (*n* = 5), and more generally, issues of access and awareness (*n* = 1) and costs (*n* = 2). While provider training for cervical cancer screening is slowly improving, multidisciplinary clinicians (pathologist, oncologists, radiologists) for the treatment of advanced cancer are far too few to meet the current demand [92, 237]. It’s important to note that many of the tertiary prevention articles also addressed feasibility concerns in the discussion sections, but this subsection reflects articles with a primary focus on feasibility.

The majority of research on tertiary prevention for cervical cancer was conducted in South Africa and Nigeria, yet research from countries in West, East and Southern Africa were also represented. 82 % were original research articles, 11 % review articles and the remaining 7 % were a combination of reports and commentary articles.

### Quality of life

While there were a significant number of publications focusing on various aspects of quality of life among cervical cancer patients and survivors in the United States, Europe, and an increasing number from Taiwan, we found only *n* = 16 publications from countries in Africa. Publications were organized into the following areas of focus: 8 (50 %) on quality of life (physical, psychological, emotional, social, and spiritual), 5 (31 %) on palliative care, 1 (6 %) on partner support, and 2 (13 %) on symptoms and complications, see Table 4.

**Table 4:** Quality of life research, *n* = 16

Focus		Literature type	Countries/Geographic Regions Included
Quality of Life	Quality of Life Outcomes *N* = 8	6 Original research [101, 104, 118, 450–452] 2 Review Articles [103, 249] 0 Lessons Learned 0 Policy Papers	South Africa (2) [450, 451], Kenya (1) [104], Nigeria (2) [101, 118], Sudan (1) [452], Global-12 world regions (1) [103], Uganda (3) [249, 450, 451]
	Palliative Care *N* = 5	3 Original research [250, 252, 253] 1 Review Articles [251] 0 Lessons Learned 1 Policy [117]	Nigeria (1) [250], sub-Saharan Africa (1) [253], Africa (2) [117, 251], South Africa (1) [252], Uganda (1) [252]
	Partner Support *N* = 1	1 Original research [108] 0 Review Articles 0 Lessons Learned 0 Policy	South Africa (1) [108]
	Symptoms and complications *N* = 2	2 Original research [107, 254] 0 Review Articles 0 Lessons Learned 0 Policy	Nigeria (1) [107], Uganda (1) [254]
	Total *N* = 16	12 Original research 3 Review Articles 0 Lessons Learned 1 Policy Paper	Global (1), SSA (3), South Africa (4), Kenya (1), Nigeria (4), Sudan (1), Uganda (5)

Among the seven publications describing quality of life outcomes, five studies reported the patient perspective and 1 study included data from both cervical cancer patients and caregivers. In regard to the timing of quality of life assessments, approximately half of these research studies included patients actively receiving treatment (typically radiotherapy) and the other half were receiving palliative care. A recent review article estimated age-adjusted, daily-adjusted life years lost attributed to various types of cancer, including cervical cancer, in sub-Saharan Africa and other global regions. Five studies focused on palliative care for cancer patients (specific to or including cervical cancer patients) in Africa highlighting challenges to provide adequate pain management [117, 249, 250], and training to optimize palliative care for cancer patients [251, 252], including one review article [253]. Only one study directly addressed partner support for cervical cancer patients [108]. One study assessed urologic complications among women with advanced cervical cancer in Uganda prior to treatment [254], and another assessed Cisplatin based chemotherapy for treatment of long term vaginal bleeding [107]. The majority of these studies was conducted in Nigeria, South Africa, and Uganda or had a sub-Saharan Africa regional focus.

Despite the heavy burden of disease and limited access to cervical cancer treatment, research regarding multiple quality of life issues for women, their partners, and caregivers is still limited for countries in Africa. A notable gap in the literature is attention to fertility-related concerns and the impact of the disease and its treatment on women’s bodies, sexual function, and relationships.

### Distribution of cervical cancer research in Africa

**Fig. 2:**
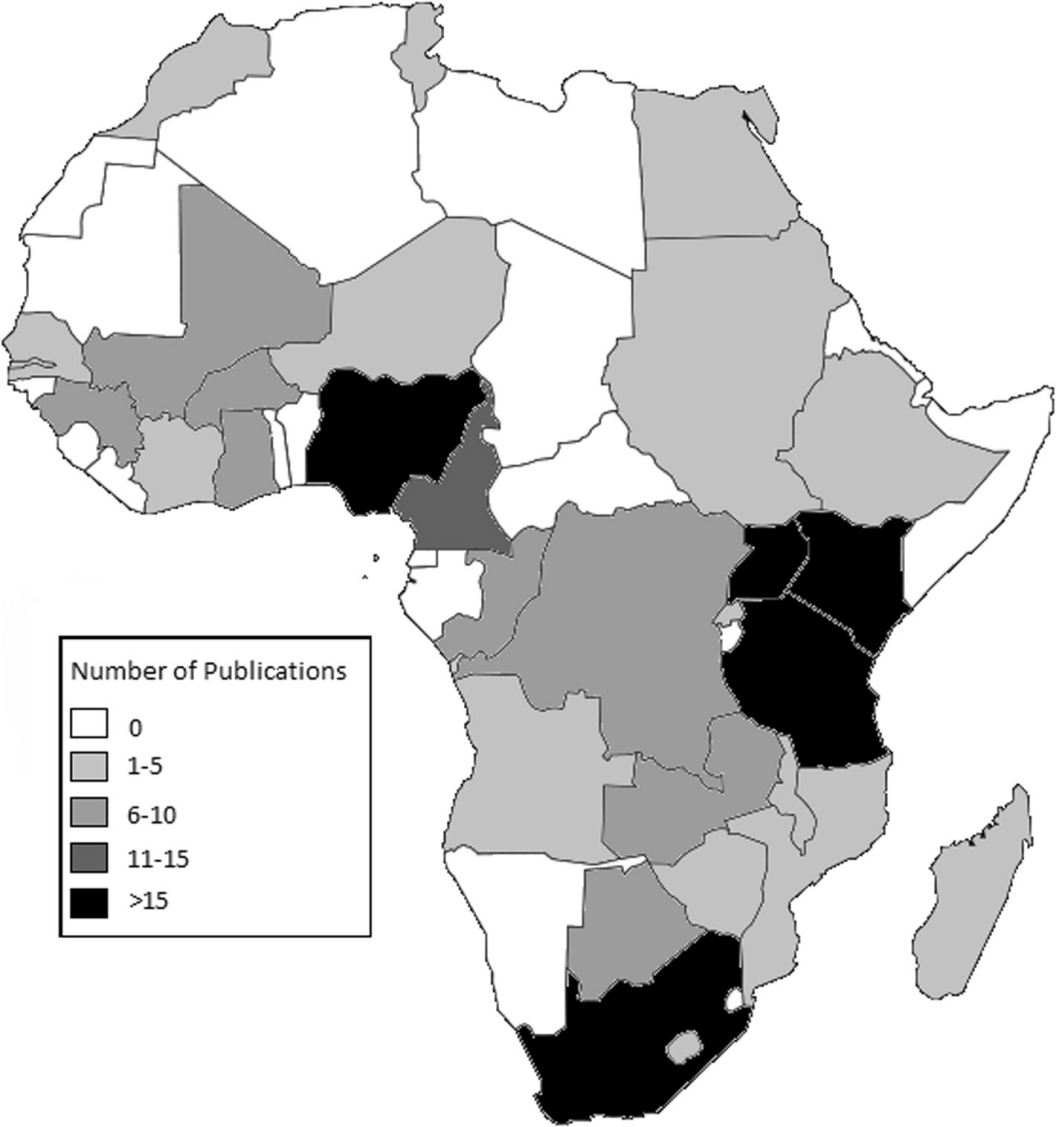
Geographic Distribution of Cervical Cancer Research within Africa. Figure 2 is a map of Africa illustrating the geographic distribution of cervical cancer research. The map represents individual countries only and does not clearly illustrate some of the smaller African countries. Fifty articles reporting on a geographic region were excluded (Excluded articles reported on the following geographic regions: Middle East/North Africa (2); 3 GAVI-eligible countries (3); Global (6); LMIC countries/developing countries (14); Africa/Sub-Saharan Africa (25). Image modified from: Tourbillon (Own work) [Public domain], via Wikimedia Commons, available at https://commons.wikimedia.org/wiki/File:Colored_map_of_Africa.png#

Eligible articles and reports either focused globally (*n* = 4), on low or middle income countries (LMIC) (*n* = 14), the sub Saharan/African region (*n* = 27), and targeted multiple (*n* = 27) or single (*n* = 308) countries in Africa. A total of *n* = 30 countries in Africa were represented by at least one publication. Figure 2 illustrates the density of cervical cancer research publications by country. This excludes publications with a regional focus. Five categories ranging from 0 to >15 publications are delineated by a color gradient per country.

South Africa had the highest number of cervical cancer publications included in this review (*n* = 85), followed by Nigeria (*n* = 61), and Uganda and Kenya, with 33 and 32 publications, respectively. These same four countries also have cervical cancer publications representing each of the four target categories: primary, secondary, tertiary prevention and quality of life. Botswana, Cote d’Ivoire, Malawi and Morocco had publications from each of the three types of prevention, but none related to quality of life. Eligible cervical cancer publications were missing from a total of 24 African countries, including: Algeria, Benin, Burundi, Cape Verde, Central African Republic, Chad, Comoros, Djibouti, Equatorial Guinea, Eritrea, Gabon, Guinea-Bissau, Liberia, Libya, Mauritania, Mauritius, Namibia, Sao Tome and Principe, Seychelles, Sierra Leone, Somalia, South Sudan, Swaziland, and Togo.

## Discussion

This study reviewed cervical cancer related research from countries in Africa over the past decade and categorized them according to primary focus areas within public health. Among a total of 380 research articles/reports eligible for inclusion in this systematic review, the majority (54.6 %) focused on secondary prevention. The number of publication focusing on primary prevention (23.4 %), particularly HPV vaccination, increased significantly in the past decade. Research regarding the treatment of precancerous lesions and invasive cervical cancer are emerging (17.6 %), but infrastructure and feasibility challenges in many countries have impeded efforts to provide and evaluate such treatment services. Studies assessing varying aspects of quality of life among women living with cervical cancer are limited, representing only 4.1 % of eligible publications. Across all categories, 11.3 % of publications focused on cervical cancer prevention or treatment among HIV-infected women. While integrated throughout, *n* = 65 (17.1 %) publications focused on various aspects of feasibility for cervical cancer control efforts, appealing for increased financial, human and political investments to adequately address the existing and increasing need for cervical cancer prevention and treatment services.

### Limitations

While a wide range of articles are referenced in the introduction of this manuscript, the results section of the review excludes a significant number of articles describing the epidemiology of cervical cancer in sub Saharan Africa or a focus on genetic biomarkers, which were beyond the scope of this review. Some publications had significant overlap in the topics presented. This was particularly true for findings on secondary and tertiary prevention. For the purpose of this review, publications were assigned to only one category, but this does not mean other articles or reports did not give a degree of focus to additional categories as well.

## Conclusion

The number of publications focused on cervical cancer control and treatment in African countries has increased over the past decade. Tertiary prevention (i.e. treatment of disease with effective medicine) and quality of life of cervical cancer survivors are two under-researched areas in African countries. Similarly, there are several countries in Africa with little to no research ever conducted on cervical cancer. Given the significant burden and high morbidity and mortality experienced by women with cervical cancer in this setting, targeted research is critically needed, particularly implementation science research to inform feasible and sustainable strategies to maximize the number of women reached with services. Lessons learned reports and publications describing and evaluating service implementation are highly relevant, as are cost-effectiveness studies to guide service strategies for scale-up. There is a need to support national capacity for developing population-based cancer registries to track cases and project needs [255], strategies to increase the availability of cervical cancer screening and treatment, and innovative strategies to retain cervical cancer patient in treatment.

## Abbreviations

AFCRN, African Cancer Registry Network; AIDS, autoimmune deficiency syndrome; AIM, African index medicus; AORTIC, African Organization for Research and Training Center; CIN, cervical intraepithelial neoplasia; DALY, daily adjusted life years; DNA, deoxyribonucleic acid; GAVI, global alliance for vaccines and immunizations; HIV, human immunodeficiency virus; HPV, human papilloma virus; LEEP, loop electrical excision procedure; LEETZ, large loop excision of the transformation zone; LMIC, low and middle income countries; NCBI, National Center for Biotechnology Information; SSA, Sub Saharan Africa; VIA, visual inspection with acetic acid; VILI, visual inspection with lugol’s iodine; WHO, World Health Organization
